# Aerobic running at different intensities produces distinct temporal patterns of improvement in depression, anxiety, and stress among adolescents with problematic internet use

**DOI:** 10.3389/fpsyg.2026.1769651

**Published:** 2026-06-26

**Authors:** Zhanpeng Meng, Chuanwen Yu, Yihao Li, Yanling Hu, Xiangying Wang

**Affiliations:** 1 College of Physical Education, Shandong Normal University, Jinan, Shandong, China; 2 College of Physical Education and Health, Heze University, Heze, Shandong, China; 3 College of Foreign Languages, Shandong Normal University, Jinan, Shandong, China

**Keywords:** aerobic running, anxiety, depression, exercise intensity, negative emotion, problematic internet use

## Abstract

**Objective:**

To examine the effects of low-, moderate-, and high-intensity aerobic running on depression, anxiety, and stress among adolescents with problematic internet use (PIU).

**Methods:**

A randomized controlled design was used. A total of 120 adolescents with PIU, aged 12–15 years, were randomly assigned to a low-intensity group, a moderate-intensity group, a high-intensity group, or a control group (*n* = 30 per group). The intervention lasted 12 weeks. Exercise intensity was defined according to percentages of maximal heart rate (HRmax): low intensity, 40–49% HRmax; moderate intensity, 50–59% HRmax; and high intensity, 60–69% HRmax. Depression, anxiety, and stress were assessed at pretest, midtest, and posttest using the Depression Anxiety Stress Scales-21 (DASS-21). Repeated-measures mixed-effects models were fitted for each outcome, followed by Bonferroni-adjusted simple-effects analyses.

**Results:**

Significant main effects of group and time, as well as significant group × time interactions, were observed for depression, anxiety, and stress. Compared with the control group, all three exercise conditions showed lower depression, anxiety, and stress scores at posttest. No statistically significant posttest differences were found among the three intervention groups. However, the temporal patterns of improvement differed across exercise intensities. Moderate-intensity running showed a more continuous pattern of change across the intervention period, high-intensity running showed earlier improvement, particularly at midtest, and low-intensity running also produced significant benefits, although its effects emerged later.

**Conclusion:**

Aerobic running can reduce depression, anxiety, and stress among adolescents with PIU. The findings suggest that intensity-related differences are better understood in terms of timing and trajectory of emotional change rather than endpoint superiority across exercise conditions. These findings provide intensity-specific evidence to inform more targeted exercise prescriptions for adolescents with PIU.

## 1 Introduction

Adolescence is a critical period for psychological and social development. Mental health problems during this stage are common and consequential. Globally, one in seven adolescents aged 10–19 years experiences a mental disorder, and depression and anxiety are among the leading causes of illness and disability in this age group (World Health Organization, 2025). In China, recent meta-analytic evidence indicates that depressive symptoms are also highly prevalent among children and adolescents (Zhou J. et al., 2024). Therefore, identifying effective and scalable strategies to reduce negative emotions in adolescents remains an important priority in public health and education.

Among the many factors associated with adolescent mental health, problematic internet use (PIU) has received increasing attention. Adolescents are particularly vulnerable to excessive or maladaptive internet use because of the widespread availability of smartphones and online platforms. PIU may displace sleep, study, physical activity, and face-to-face interaction. It may also increase social comparison, emotional dependence on online feedback, and difficulties in self-regulation (Weinstein and Lejoyeux, 2010; Kuss and Griffiths, 2012). Previous research has also linked PIU to altered reward processing and reduced cognitive control, particularly in prefrontal systems involved in impulse regulation and emotion regulation (León Méndez et al., 2024). Recent review evidence further suggests a predominantly bidirectional relationship: emotional distress increases the risk of PIU, and PIU in turn aggravates anxiety, depression, and emotional dysregulation in adolescents (Villanueva-Blasco et al., 2026). Taken together, these findings suggest that interventions for adolescents with PIU need to address both internet-related behaviors and negative emotions simultaneously.

Physical exercise, especially aerobic exercise, has been widely recognized as a promising non-pharmacological strategy for improving mental health. Exercise may reduce negative emotions through both biological and psychosocial pathways (Lubans et al., 2016). Recent evidence also suggests that exercise-based interventions can reduce internet addiction symptoms in adolescents (Wang et al., 2025). However, two important gaps remain. First, findings on exercise intensity are still inconsistent, with some studies favoring moderate intensity and others reporting stronger short-term effects for high intensity. Second, few studies have specifically focused on adolescents with PIU and directly compared different exercise intensities of the same aerobic exercise while tracking changes in depression, anxiety, and stress across multiple time points. As a result, it remains unclear which intensity profile is most appropriate for this population and whether intensity mainly influences the magnitude of improvement, the timing of improvement, or both.

To address these gaps, the present study compared the effects of low-, moderate-, and high-intensity aerobic running in adolescents with PIU. A blank control group was also included. Depression, anxiety, and stress were assessed at pretest, midtest, and posttest across a 12-week intervention period. The study had two main objectives. First, it aimed to determine whether aerobic running at different intensities could improve negative emotions relative to a non-exercise control condition. Second, it aimed to examine whether different exercise intensities produced different temporal patterns of emotional change. By clarifying these issues, the study sought to provide evidence for more targeted exercise prescriptions for adolescents with PIU.

## 2 Methods

### 2.1 Research design and participants

The present study used a randomized experimental design to examine how different intensities of aerobic running influenced negative emotions in adolescents with Problematic Internet Use (PIU). The formal intervention lasted 12 weeks and included three exercise groups, namely low-, moderate-, and high-intensity aerobic running, as well as a blank control group that did not participate in any structured physical activity. The control group was included to reflect natural emotional changes in the absence of intervention and to provide a baseline for comparison. Emotional data were collected at three time points: before the intervention (T1), at the midpoint of the intervention (T2, week 6), and after the intervention (T3, week 12). To ensure consistency in administration, all questionnaires were completed collectively in the classroom under standardized supervision.

Participants were 120 adolescents aged 12–15 years, recruited from Middle School A in Jining City, Shandong Province, China. All participants were screened using the Chinese version of Young’s Internet Addiction Test (IAT), and those with scores ≥50 were identified as exhibiting PIU. The inclusion criteria were as follows: (1) meeting the PIU threshold on the IAT; (2) having no major physical or psychological illness; (3) having the basic physical capacity to participate in exercise; and (4) providing written informed consent from both the participant and the legal guardian. The exclusion criteria were: (1) a history of cardiovascular, respiratory, or musculoskeletal disorders that contraindicated exercise; (2) current use of psychotropic medication; and (3) a clinical diagnosis of severe mental disorder.

After screening, 120 eligible participants were enrolled in the formal experiment and assigned to the four groups using simple randomization in a 1:1:1:1 ratio. The random allocation sequence was generated using Excel-generated random numbers, and participants were assigned accordingly to the low-intensity, moderate-intensity, high-intensity, and control groups (n = 30 per group). All four groups were assessed at T1, T2, and T3. The study protocol was approved by the Ethics Committee of the College of Physical Education of Shandong Normal University (Approval No. SDNUTYDW2025008). Written informed consent was obtained from all participants and their guardians before participation. The study was conducted in accordance with the Declaration of Helsinki.

The total study period lasted 13 weeks. The first week was used for questionnaire administration and a pilot phase. The pilot phase was conducted before the formal experiment and was used only to refine the intervention protocol. Data from the pilot phase were not included in the final analysis. The formal intervention began on April 7, 2025, and ended on June 27, 2025, after 12 consecutive weeks of training. Before the intervention, we predefined that participants who missed more than three exercise sessions would be excluded from the final analysis; however, no participant exceeded this threshold during the formal experiment. Although the control group did not receive a structured exercise intervention during the 12-week study period, all participants remained under routine school supervision. Class teachers and school staff were aware of the study and could observe participants’ general emotional status during the study period. Participation was voluntary, and both participants and their legal guardians were informed that withdrawal was permitted at any time. If any participant had shown marked emotional deterioration or requested help, the case would have been communicated to the guardian and referred to the appropriate school support channel.

### 2.2 Intervention protocol

Based on previous research, aerobic running intensity was divided into three levels: low, moderate, and high. Participants in all three groups were required to complete 30 min of aerobic running per session. According to prior studies, the maximum heart rate (HRmax) was calculated using the formula *HRmax = 220 − age*. Exercise intensity for each group was determined by the participant’s heart rate: low intensity corresponded to 40%–49% of HRmax (target 45%), moderate intensity to 50–59% of HRmax (target 55%), and high intensity to 60%–69% of HRmax (target 65%) (Jeon and Ha, 2017). Heart rate was continuously monitored using a Polar heart rate monitor. Each aerobic running session lasted 40 min in total, consisting of a 5-min warm-up phase, 30 min of running at the target intensity, and a 5-min cool-down phase for relaxation and recovery. Heart rate data were recorded throughout the session, and the session record could be reviewed after exercise as a reference for the implementation of the prescribed intensity.

### 2.3 Negative emotion measurement

In this study, adolescents’ negative emotions were assessed using the Depression Anxiety Stress Scale-21 (DASS-21), which measures three dimensions: depression, anxiety, and stress. The scale, developed by Lovibond and Lovibond (1995), consists of 21 items, with seven items per dimension. Participants rated each item based on their experiences during the past week using a 4-point Likert scale ranging from 0 (“did not apply to me at all”) to 3 (“applied to me very much or most of the time”). Scores for each subscale were added together and then doubled to produce the final results, with higher totals reflecting more severe emotional symptoms. The Chinese adaptation of the DASS-21 has been widely used among adolescents and shown solid psychometric properties, including Cronbach’s *α* values exceeding 0.85 across all dimensions(Wang et al., 2016; Luo et al., 2025). In this study, participants completed the DASS-21 on three occasions—prior to the intervention, halfway through the program, and upon its completion—allowing for a dynamic assessment of changes in depression, anxiety, and stress throughout the 12-week period.

### 2.4 Problematic internet use

Adolescents’ problematic internet use was evaluated with Young’s Internet Addiction Test (YIAT), originally developed by Kimberly Young in 1998. The questionnaire contains 20 items that examine four key aspects of online behavior: compulsive engagement, withdrawal tendencies, tolerance development, and the resulting functional disruptions in daily life. Each item is rated on a 5-point Likert scale ranging from 1 (“rarely”) to 5 (“always”), yielding a total score between 20 and 100. Higher scores indicate more severe Internet use problems. The Chinese version of the IAT, which has been validated among adolescent populations in China, demonstrates good reliability and validity, with Cronbach’s *α* coefficients typically above 0.85 and satisfactory construct validity (Lai et al., 2013; Li, 2022). In accordance with previous research, this study adopted a total score of 50 or above as the cutoff criterion for identifying Problematic Internet Use. In the present study, the IAT was administered only at baseline to identify adolescents who met the criterion for PIU and to define the study population. It was not included as a repeated longitudinal outcome measure.

### 2.5 Statistical analysis

Statistical analyses were conducted in R. First, Harman’s single-factor test was used to assess the potential influence of common method bias. Next, normality and homogeneity of variance were tested. One-way analysis of variance (ANOVA) was then used to examine baseline differences in internet addiction severity across groups. Repeated-measures mixed-effects models were fitted separately for depression, anxiety, and stress. In these models, group, time, and the group × time interaction were treated as fixed effects, and participant was treated as a random effect. Estimated marginal means (EMMs) and their 95% confidence intervals were calculated for each group at each time point. When a significant interaction was found, Bonferroni-adjusted simple-effects analyses were performed to compare between-group differences at each time point and within-group differences across time points. Statistical significance was set at *p* < 0.05.

## 3 Results

All 120 participants who entered the formal experiment completed the intervention and were included in the final analysis. No participant exceeded the predefined absence threshold during the intervention period. This study used the Young’s Internet Addiction Test (YAIT) and the Depression Anxiety Stress Scale-21 (DASS-21) as the primary assessment tools. To assess the potential influence of common method bias, Harman’s single-factor test was conducted prior to formal data analysis. All items from the two scales were entered into an exploratory factor analysis to examine potential common method bias. The analysis showed that the first unrotated factor accounted for 13.99% of the total variance in the YAIT and 17.91% in the DASS-21. Because both values were below the 40% threshold, common method variance was considered unlikely to pose a serious concern, and the data were deemed suitable for subsequent analyses. Baseline comparisons of internet addiction levels were also conducted across the four groups before the intervention. Tests of normality and homogeneity of variance indicated that the data met the assumptions required for parametric analyses. A Kruskal–Wallis H test revealed no significant between-group differences in baseline depression, anxiety, or stress scores (all *p* > 0.05), indicating that the four groups were comparable at baseline in terms of negative emotion severity. This baseline equivalence strengthened confidence in the validity of the subsequent analyses (see Table 1).

**Table 1:** Group-level medians (interquartile ranges) for depression, anxiety, and stress at pretest and between-group comparisons.

Negative emotions	Control, Median (Q1, Q3)	Low-Median (Q1, Q3)	Moderate-Median (Q1, Q3)	High-Median (Q1, Q3)	H	df	*p*
Depression	6.50 (5.00, 8.00)	6.50 (5.00, 8.00)	6.00 (4.00, 8.00)	6.50 (5.00, 8.00)	0.179	3	0.981
Anxiety	7.00 (6.00, 14.00)	7.00 (6.00, 12.00)	6.00 (6.00, 13.00)	6.50 (6.00, 12.00)	0.509	3	0.917
Stress	12.50 (8.00, 16.00)	13.00 (10.00, 16.00)	12.50 (8.00, 15.00)	12.50(8.00, 16.00)	0.396	3	0.941

### 3.1 Overall effects of aerobic running at different intensities on the dimensions of negative emotion

Results from the repeated-measures mixed-effects model (Table 2) revealed significant main effects of group and time, as well as significant group × time interactions, for depression, anxiety, and stress. For depression, significant effects were observed for group, *F*(3, 116) = 3.392, *p* = 0.020; time, *F*(2, 232) = 90.821, *p* < 0.001; and the group × time interaction, *F*(6, 232) = 12.880, *p* < 0.001. Similarly, for anxiety, significant effects were observed for group, F(3, 116) = 4.283, *p* = 0.007; time, F(2, 232) = 63.645, *p* < 0.001; and the group × time interaction, F(6, 232) = 10.312, *p* < 0.001. A similar pattern was found for stress, with significant effects of group, F(3, 116) = 6.388, *p* < 0.001; time, F(2, 232) = 157.875, *p* < 0.001; and the group × time interaction, F(6, 232) = 27.253, *p* < 0.001. Taken together, these findings indicate that the trajectories of depression, anxiety, and stress differed across groups over time, thereby justifying follow-up simple-effects analyses.

**Table 2:** Tests of fixed effects for the dimensions of negative emotions.

Negative emotions	Fixed effects	F	Num DF	Den DF	P
Depression	Group	3.392	3	116	0.020
	Time	90.821	2	232	<0.001
	Group × Time	12.880	6	232	<0.001
Anxiety	Group	4.283	3	116	0.007
	Time	63.645	2	232	<0.001
	Group × Time	10.312	6	232	<0.001
Stress	Group	6.388	3	116	<0.001
	Time	157.875	2	232	<0.001
	Group × Time	27.253	6	232	<0.001

NumDF, numerator degrees of freedom; DenDF, denominator degrees of freedom. *p* values were adjusted using the Bonferroni method.

### 3.2 Patterns of change in the dimensions of negative emotion across groups at different time points

The estimated marginal means (EMMs) indicated that scores were generally comparable across groups at pretest for all three dimensions of negative emotion, suggesting similar baseline levels. As the intervention progressed, scores for depression, anxiety, and stress declined in all three exercise groups, whereas those in the control group remained relatively stable (Table 3). For depression, the EMMs were 7.367, 7.300, 7.400, and 7.667 at pretest; 6.367, 5.167, 3.950, and 7.500 at midtest; and 2.900, 2.083, 3.233, and 7.567 at posttest for the low-, moderate-, high-intensity, and control groups, respectively. By posttest, all three intervention groups showed lower depression scores than the control group, with the lowest mean observed in the moderate-intensity group. For anxiety, the corresponding EMMs were 8.933, 8.867, 8.933, and 9.533 at pretest; 8.400, 6.600, 5.083, and 9.500 at midtest; and 3.667, 2.833, 4.333, and 9.500 at posttest. The high-intensity group showed the largest reduction at midtest, whereas the moderate-intensity group had the lowest posttest mean. For stress, the corresponding EMMs were 12.567, 12.000, 12.533, and 12.833 at pretest; 11.567, 9.433, 8.017, and 12.800 at midtest; and 6.000, 5.000, 7.300, and 13.000 at posttest. Overall, stress scores declined in all three intervention groups, with the lowest posttest mean observed in the moderate-intensity group; the high-intensity group showed the greatest reduction at midtest but less additional improvement from midtest to posttest.

**Table 3:** Estimated marginal means of the dimensions of negative emotions at different time point.

Negative emotions	Group	Time	EMM	SE	Lower 95% CI	Upper 95% CI
Depression	Low	Pre	7.367	0.760	5.865	8.869
	Moderate	Pre	7.300	0.760	5.798	8.802
	High	Pre	7.400	0.760	5.898	8.902
	Control	Pre	7.667	0.760	6.165	9.169
	Low	Mid	6.367	0.760	4.865	7.869
	Moderate	Mid	5.167	0.760	3.665	6.669
	High	Mid	3.950	0.760	2.448	5.452
	Control	Mid	7.500	0.760	5.998	9.002
	Low	Post	2.900	0.760	1.398	4.402
	Moderate	Post	2.083	0.760	0.581	3.585
	High	Post	3.233	0.760	1.731	4.735
	Control	Post	7.567	0.760	6.065	9.069
Anxiety	Low	Pre	8.933	0.879	7.200	10.667
	Moderate	Pre	8.867	0.879	7.133	10.600
	High	Pre	8.933	0.879	7.200	10.667
	Control	Pre	9.533	0.879	7.800	11.267
	Low	Mid	8.400	0.879	6.667	10.133
	Moderate	Mid	6.600	0.879	4.867	8.333
	High	Mid	5.083	0.879	3.350	6.817
	Control	Mid	9.500	0.879	7.767	11.233
	Low	Post	3.667	0.879	1.933	5.400
	Moderate	Post	2.833	0.879	1.100	4.567
	High	Post	4.333	0.879	2.600	6.067
	Control	Post	9.500	0.879	7.200	10.667
Stress	Low	Pre	12.567	0.782	11.022	14.111
	Moderate	Pre	12.000	0.782	10.456	13.544
	High	Pre	12.533	0.782	10.989	14.078
	Control	Pre	12.833	0.782	11.289	14.378
	Low	Mid	11.567	0.782	10.022	13.111
	Moderate	Mid	9.433	0.782	7.889	10.978
	High	Mid	8.017	0.782	6.472	9.561
	Control	Mid	12.800	0.782	11.256	14.344
	Low	Post	6.000	0.782	4.456	7.544
	Moderate	Post	5.000	0.782	3.456	6.544
	High	Post	7.300	0.782	5.756	8.844
	Control	Post	13.000	0.782	11.456	14.544

EMM, estimated marginal mean; SE, standard error; Lower 95% CI, lower bound of the 95% confidence interval; Upper 95% CI, upper bound of the 95% confidence interval. Low, low-intensity aerobic running group; Moderate, moderate-intensity aerobic running group; High, high-intensity aerobic running group; Control, blank control group; Pre, pre-intervention; Mid, mid-intervention; Post, post-intervention. *p* values were adjusted using the Bonferroni method.

### 3.3 Between-group simple-effects comparisons for the dimensions of negative emotion at different time points

To further examine between-group differences at each assessment point, simple-effects comparisons were conducted separately for depression, anxiety, and stress, with Bonferroni-adjusted *p* values reported throughout. For depression, Table 4 shows that no significant between-group differences were observed at pretest (all *p* > 0.05), further supporting baseline comparability across groups. At midtest, only the high-intensity group scored significantly lower than the control group (MD = −3.550, *p* = 0.007), whereas no other pairwise comparisons reached statistical significance. At posttest, the low-, moderate-, and high-intensity groups all showed significantly lower depression scores than the control group (MDs = −4.667, −5.483, and −4.333, respectively, all *p* < 0.001), whereas no significant differences were found among the three intervention groups (all *p* > 0.05). These findings indicate that, by the end of the intervention, all three exercise conditions were associated with lower depression scores than the control condition, although no significant between-intervention differences emerged.

**Table 4:** Between-group simple effects comparisons for the dimensions of negative emotions at different time points.

Negative Emotions	Time	BGC	MD	SE	df	t	*p*
Depression	Pre	Low - Moderate	0.067	1.075	159.581	0.062	1.000
	Pre	Low - High	−0.033	1.075	159.581	−0.031	1.000
	Pre	Low - Control	−0.300	1.075	159.581	−0.279	1.000
	Pre	Moderate - High	−0.100	1.075	159.581	−0.093	1.000
	Pre	Moderate-Control	−0.367	1.075	159.581	−0.341	1.000
	Pre	High - Control	−0.267	1.075	159.581	−0.248	1.000
	Mid	Low - Moderate	1.200	1.075	159.581	1.116	1.000
	Mid	Low - High	2.417	1.075	159.581	2.247	0.156
	Mid	Low - Control	−1.133	1.075	159.581	−1.054	1.000
	Mid	Moderate - High	1.217	1.075	159.581	1.131	1.000
	Mid	Moderate - Control	−2.333	1.075	159.581	−2.170	0.189
	Mid	High - Control	−3.550	1.075	159.581	−3.301	0.007
	Post	Low - Moderate	0.817	1.075	159.581	0.759	1.000
	Post	Low - High	−0.333	1.075	159.581	−0.310	1.000
	Post	Low - Control	−4.667	1.075	159.581	−4.339	<0.001
	Post	Moderate - High	−1.150	1.075	159.581	−1.069	1.000
	Post	Moderate - Control	−5.483	1.075	159.581	−5.099	<0.001
	Post	High - Control	−4.333	1.075	159.581	−4.029	<0.001
Anxiety	Pre	Low - Moderate	0.067	1.242	182.283	0.054	1.000
	Pre	Low - High	0.000	1.242	182.283	0.000	1.000
	Pre	Low - Control	−0.600	1.242	182.283	−0.483	1.000
	Pre	Moderate - High	−0.067	1.242	182.283	−0.054	1.000
	Pre	Moderate - Control	−0.667	1.242	182.283	−0.537	1.000
	Pre	High - Control	−0.600	1.242	182.283	−0.483	1.000
	Mid	Low - Moderate	1.800	1.242	182.283	1.449	0.895
	Mid	Low - High	3.317	1.242	182.283	2.669	0.050
	Mid	Low - Control	−1.100	1.242	182.283	−0.885	1.000
	Mid	Moderate - High	1.517	1.242	182.283	1.221	1.000
	Mid	Moderate - Control	−2.900	1.242	182.283	−2.334	0.124
	Mid	High - Control	−4.417	1.242	182.283	−3.555	0.003
	Post	Low - Moderate	0.833	1.242	182.283	0.671	1.000
	Post	Low - High	−0.667	1.242	182.283	−0.537	1.000
	Post	Low - Control	−5.833	1.242	182.283	−4.695	<0.001
	Post	Moderate - High	−1.500	1.242	182.283	−1.207	1.000
	Post	Moderate - Control	−6.667	1.242	182.283	−5.366	<0.001
	Post	High - Control	−5.167	1.242	182.283	−4.158	<0.001
Stress	Pre	Low - Moderate	0.567	1.106	158.323	0.512	1.000
	Pre	Low - High	0.033	1.106	158.323	0.030	1.000
	Pre	Low - Control	−0.267	1.106	158.323	−0.241	1.000
	Pre	Moderate - High	−0.533	1.106	158.323	−0.482	1.000
	Pre	Moderate - Control	−0.833	1.106	158.323	−0.754	1.000
	Pre	High - Control	−0.300	1.106	158.323	−0.271	1.000
	Mid	Low - Moderate	2.133	1.106	158.323	1.929	0.333
	Mid	Low - High	3.550	1.106	158.323	3.210	0.010
	Mid	Low - Control	−1.233	1.106	158.323	−1.115	1.000
	Mid	Moderate - High	1.417	1.106	158.323	1.281	1.000
	Mid	Moderate - Control	−3.367	1.106	158.323	−3.044	0.016
	Mid	High - Control	−4.783	1.106	158.323	−4.325	<0.001
	Post	Low - Moderate	1.000	1.106	158.323	0.904	1.000
	Post	Low - High	−1.300	1.106	158.323	−1.176	1.000
	Post	Low - Control	−7.000	1.106	158.323	−6.330	<0.001
	Post	Moderate - High	−2.300	1.106	158.323	−2.080	0.235
	Post	Moderate - Control	−8.000	1.106	158.323	−7.234	<0.001
	Post	High - Control	−5.700	1.106	158.323	−5.154	<0.001

BGC, between-group comparisons; MD, mean difference; SE, standard error; df, degrees of freedom; Low, low-intensity aerobic running group; Moderate, moderate-intensity aerobic running group; High, high-intensity aerobic running group; Control, blank control group; Pre, pre-intervention; Mid, mid-intervention; Post, post-intervention. *p* values were adjusted using the Bonferroni method.

For anxiety, Table 4 shows that no significant between-group differences were found at pretest (all *p* > 0.05). At midtest, only the high-intensity group scored significantly lower than the control group (MD = −4.417, *p* = 0.003). At posttest, the low-, moderate-, and high-intensity groups all showed significantly lower anxiety scores than the control group (MDs = −5.833, −6.667, and −5.167, respectively, all *p* < 0.001), whereas no significant differences were observed among the three intervention groups (all *p* > 0.05). These findings indicate that, for anxiety, all three intervention intensities were associated with lower posttest scores relative to the control group, whereas the intervention groups did not differ significantly from one another.

For stress, Table 4 shows that no significant between-group differences were observed at pretest (all *p* > 0.05). At midtest, the high-intensity group differed significantly from both the low-intensity group (MD = 3.550, *p* = 0.010) and the control group (MD = −4.783, *p* < 0.001), with lower stress scores in the high-intensity group; the moderate-intensity group also scored significantly lower than the control group (MD = −3.367, *p* = 0.016). At posttest, the low-, moderate-, and high-intensity groups all showed significantly lower stress scores than the control group (MDs = −7.000, −8.000, and −5.700, respectively, all *p* < 0.001), whereas no significant differences were found among the three intervention groups (all *p* > 0.05). Overall, between-group differences in stress became evident at midtest, and by posttest all three exercise interventions were associated with lower stress scores than the control condition.

### 3.4 Within-group simple-effects comparisons for the dimensions of negative emotion across time

To further clarify within-group changes across the pretest, midtest, and posttest assessments, simple-effects comparisons over time were conducted separately for depression, anxiety, and stress in each of the four groups. For depression, no significant difference was found between pretest and midtest in the low-intensity group (*p* = 0.164), whereas both the pretest-posttest and midtest-posttest comparisons were significant (both *p* < 0.001), indicating that the reduction in depression emerged primarily during the later stage of the intervention. In the moderate-intensity group, all three pairwise comparisons across time were significant (all *p* < 0.001), suggesting a continuous decline across the intervention period. In the high-intensity group, the pretest-midtest and pretest-posttest comparisons were significant (both *p* < 0.001), whereas the midtest-posttest comparison was not (*p* = 0.503), indicating that most of the reduction occurred during the first half of the intervention. In the control group, no significant differences were observed across the three time points (all *p* > 0.05) (see Table 5).

**Table 5:** Within-group simple effects comparisons for the dimensions of negative emotions across different time points.

Negative Emotions	Group	WGC	MD	SE	df	t	*p*
Depression	Low	Pre -Mid	1.000	0.518	232	1.932	0.164
	Low	Pre -Post	4.467	0.518	232	8.629	<0.001
	Low	Mid-Post	3.467	0.518	232	6.697	<0.001
	Moderate	Pre -Mid	2.133	0.518	232	4.121	<0.001
	Moderate	Pre -Post	5.217	0.518	232	10.078	<0.001
	Moderate	Mid-Post	3.083	0.518	232	5.957	<0.001
	High	Pre -Mid	3.450	0.518	232	6.665	<0.001
	High	Pre -Post	4.167	0.518	232	8.050	<0.001
	High	Mid-Post	0.717	0.518	232	1.385	0.503
	Control	Pre -Mid	0.167	0.518	232	0.322	1.000
	Control	Pre -Post	0.100	0.518	232	0.193	1.000
	Control	Mid-Post	−0.067	0.518	232	−0.129	1.000
Anxiety	Low	Pre -Mid	0.533	0.709	232	0.752	1.000
	Low	Pre -Post	5.267	0.709	232	7.427	<0.001
	Low	Mid-Post	4.733	0.709	232	6.674	<0.001
	Moderate	Pre -Mid	2.267	0.709	232	3.196	0.005
	Moderate	Pre -Post	6.033	0.709	232	8.508	<0.001
	Moderate	Mid-Post	3.767	0.709	232	5.311	<0.001
	High	Pre -Mid	3.850	0.709	232	5.429	<0.001
	High	Pre -Post	4.600	0.709	232	6.486	<0.001
	High	Mid-Post	0.750	0.709	232	1.058	0.874
	Control	Pre -Mid	0.033	0.709	232	0.047	1.000
	Control	Pre -Post	0.033	0.709	232	0.047	1.000
	Control	Mid-Post	0.000	0.709	232	0.000	1.000
Stress	Low	Pre -Mid	1.000	0.526	232	1.902	0.175
	Low	Pre -Post	6.567	0.526	232	12.490	<0.001
	Low	Mid-Post	5.567	0.526	232	10.588	<0.001
	Moderate	Pre -Mid	2.567	0.526	232	4.882	<0.001
	Moderate	Pre -Post	7.000	0.526	232	13.314	<0.001
	Moderate	Mid-Post	4.433	0.526	232	8.432	<0.001
	High	Pre -Mid	4.517	0.526	232	8.591	<0.001
	High	Pre -Post	5.233	0.526	232	9.954	<0.001
	High	Mid-Post	0.717	0.526	232	1.363	0.523
	Control	Pre -Mid	0.033	0.526	232	0.063	1.000
	Control	Pre -Post	−0.167	0.526	232	−0.317	1.000
	Control	Mid-Post	−0.200	0.526	232	−0.380	1.000

WGC, within-group comparisons; MD, mean difference; SE, standard error; df, degrees of freedom; Low, low-intensity aerobic running group; Moderate, moderate-intensity aerobic running group; High, high-intensity aerobic running group; Control, blank control group; Pre, pre-intervention; Mid, mid-intervention; Post, post-intervention. P values were adjusted using the Bonferroni method.

For anxiety, the pretest-midtest comparison was not significant in the low-intensity group (*p* = 1.000), whereas the pretest-posttest and midtest-posttest comparisons were both significant (both *p* < 0.001). In the moderate-intensity group, all three comparisons across time reached statistical significance (pretest-midtest: *p* = 0.005; pretest-posttest and midtest-posttest: both p < 0.001). In the high-intensity group, significant differences were observed for the pretest-midtest and pretest-posttest comparisons (both *p* < 0.001), whereas the midtest-posttest comparison was not significant (*p* = 0.874). No significant changes were found across the three time points in the control group (all *p* > 0.05). Overall, anxiety scores showed a more continuous and stable pattern of improvement in the moderate-intensity group, whereas the high-intensity group appeared to improve more rapidly during the early phase of the intervention.

For stress, the pretest-midtest comparison was not significant in the low-intensity group (*p* = 0.175), whereas the pretest-posttest and midtest-posttest comparisons were both significant (both *p* < 0.001). In the moderate-intensity group, all three pairwise comparisons across time were significant (all *p* < 0.001). In the high-intensity group, the pretest-midtest and pretest-posttest comparisons were significant (both *p* < 0.001), whereas the midtest-posttest comparison was not (*p* = 0.523). No significant differences were observed across the three time points in the control group (all *p* > 0.05). Overall, the moderate-intensity group showed the most stable and sustained reduction in stress, whereas the high-intensity group exhibited an earlier response with less subsequent improvement, and the low-intensity group showed a more delayed pattern of improvement.

## 4 Discussion

This study examined the effects of a 12-week aerobic running intervention at low, moderate, and high intensities on depression, anxiety, and stress in adolescents with problematic internet use (PIU). Three main findings emerged. First, significant main effects of group and time, as well as significant group × time interactions, were observed for all three dimensions of negative emotion. This indicates that emotional trajectories differed across intervention conditions. Second, all three exercise conditions showed significant emotional improvement at posttest compared with the control condition. Third, the timing of improvement differed across exercise intensities. Moderate-intensity running showed a more continuous pattern of change across the intervention period, high-intensity running was associated with earlier improvement, and low-intensity running showed a more delayed pattern of benefit. Overall, these findings support aerobic running as an effective non-pharmacological strategy for reducing negative emotions in adolescents with PIU. At the same time, they suggest that intensity-related differences are better understood in terms of temporal trajectory rather than a simple assumption that higher intensity necessarily produces better final outcomes. This overall pattern is consistent with previous reviews and meta-analyses showing that exercise can reduce depression and anxiety in children and adolescents. It is also in line with studies reporting that sport and exercise interventions are effective for adolescent internet addiction-related problems. Wang et al. (2022) reported a moderate effect of exercise on adolescent depression and suggested that moderate intensity may be a favorable option. Singh et al. (2026) further showed in an umbrella review that exercise can moderately reduce depression and anxiety in children and adolescents. In addition, Zhou Z. et al. (2024) and Wang et al. (2025) reported in meta-analyses that sport and exercise interventions can significantly improve internet addiction-related problems in adolescents.

A particularly important finding is that all three exercise conditions outperformed the control condition at posttest, whereas no statistically significant differences were observed among the three exercise groups themselves. This pattern suggests that the main difference across exercise intensities was not the superiority of one condition over another at the endpoint, but rather the timing and trajectory of emotional change across the 12-week intervention. Descriptively, the moderate-intensity group showed the lowest posttest estimated marginal means across outcomes, whereas the high-intensity group showed earlier improvement at midtest. However, these descriptive differences should not be interpreted as evidence of statistically superior posttest efficacy for any specific exercise intensity. Instead, the present findings support a more cautious conclusion: different exercise intensities may be associated with different trajectories of emotional improvement, while all three exercise conditions were beneficial relative to the control group. This interpretation is broadly consistent with previous reviews and meta-analyses showing that exercise can improve emotional health and internet addiction-related problems in adolescents(Wang et al., 2025).

**Figure 1:**
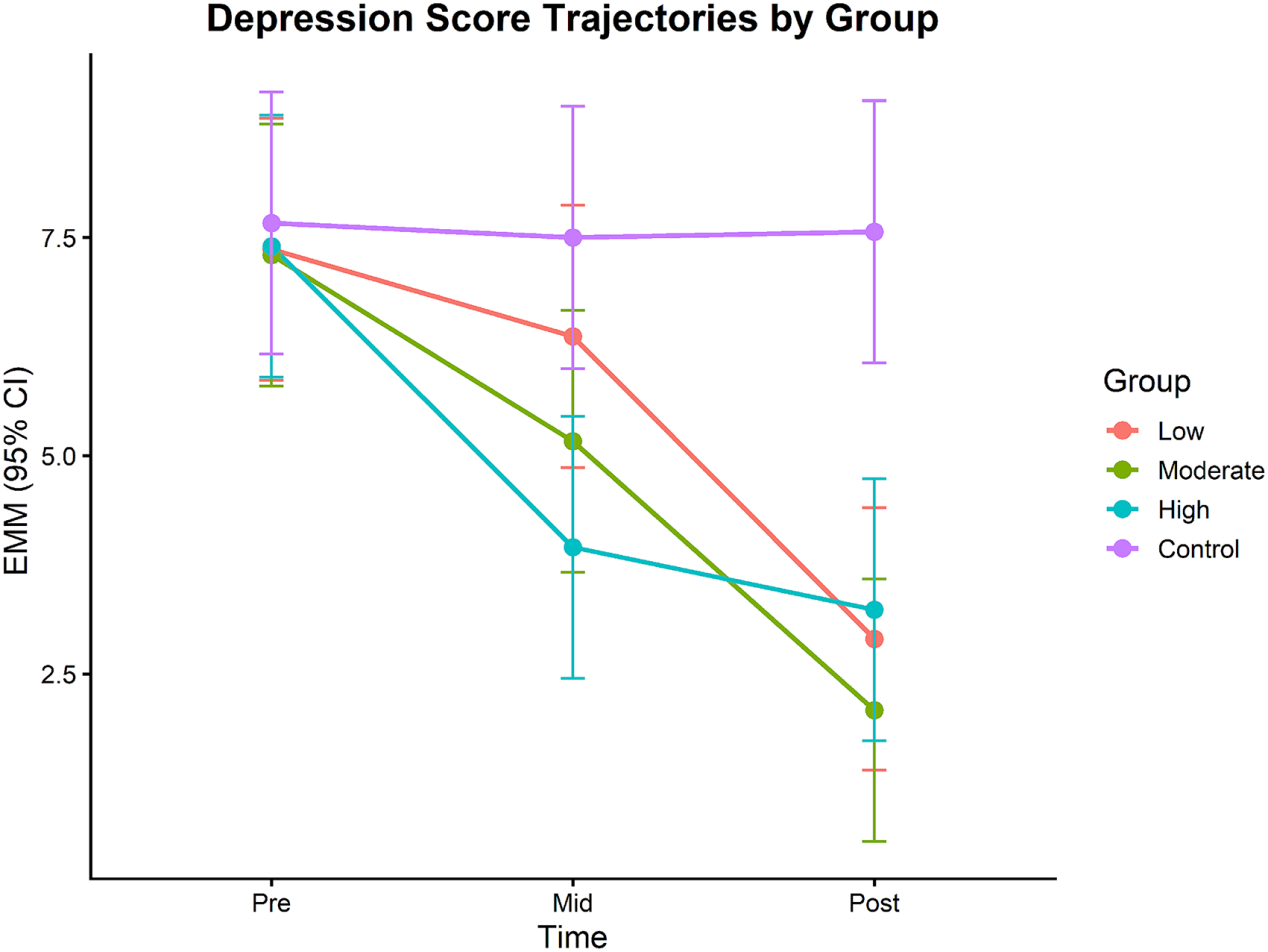
Trends in estimated marginal means of depression at different time points.

For depression (Figure 1), all three exercise conditions were superior to the control condition at posttest, but no significant differences were found among the exercise groups. The within-group simple effects further clarified the temporal pattern of change in depressive symptoms. In the low-intensity group, no significant reduction was observed from pretest to midtest. However, both the pretest-posttest and midtest-posttest comparisons were significant. This suggests that the antidepressant effect of low-intensity running developed gradually and became clearer in the later stage of the intervention. In the moderate-intensity group, all three time comparisons were significant, indicating a continuous decline in depressive symptoms across the 12-week period. In the high-intensity group, significant reductions were found from pretest to midtest and from pretest to posttest, whereas the midtest-posttest difference was not significant. This suggests that much of the improvement in depression occurred during the first half of the intervention and then stabilized. Taken together, these findings indicate that the three exercise intensities differed mainly in trajectory. Moderate-intensity running was associated with a more continuous within-group decline in depressive symptoms, whereas high-intensity running was associated with earlier improvement followed by stabilization. Because no significant posttest differences were observed among the exercise groups, these patterns should be interpreted as differences in temporal trajectory rather than evidence that one exercise intensity was definitively superior at the endpoint. This pattern is generally consistent with the findings of Wang et al. (2022) and Singh et al. (2026), both of whom suggested that exercise may be particularly beneficial for depression in young people. In addition, Hong et al. (2020) found in male adolescents with internet gaming disorder that exercise combined with CBT improved both gaming-related symptoms and depressive mood. This finding also supports the general direction of the present results.

**Figure 2:**
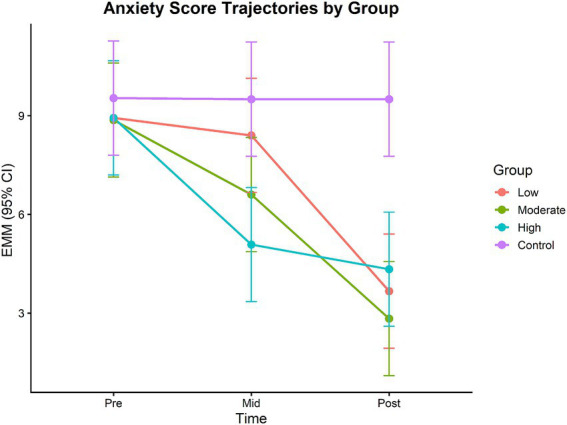
Trends in estimated marginal means of anxiety at different time points.

The findings for anxiety showed a similar (Figure 2), but not identical, pattern. At midtest, only the high-intensity group differed significantly from the control group, suggesting that a higher training load may be associated with a faster early response in anxiety reduction. However, by posttest, all three exercise conditions showed significantly lower anxiety scores than the control condition, and no significant differences were found among the exercise groups. The within-group comparisons also revealed different time patterns. In the moderate-intensity group, all three time comparisons were significant, indicating a relatively continuous and stable decline in anxiety. By contrast, the high-intensity group showed significant improvement from pretest to midtest and from pretest to posttest, but not from midtest to posttest. This means that the early gains did not increase further during the second half of the intervention. The low-intensity group also showed significant improvement by posttest, but its effects appeared later. Therefore, although high-intensity running was associated with earlier improvement in anxiety, and moderate-intensity running showed a more continuous pattern of change across time, the present data do not support the conclusion that any one exercise intensity was statistically superior to the others at posttest. At the same time, it suggests that the most suitable intensity may differ across outcome domains. For example, Ligon et al. (2025) reported a moderate effect of exercise on anxiety in children and adolescents. Song et al. (2025) further suggested that high-intensity aerobic exercise may be especially useful for anxiety management. This overall pattern remains consistent with previous evidence that exercise can reduce anxiety symptoms in children and adolescents.

**Figure 3:**
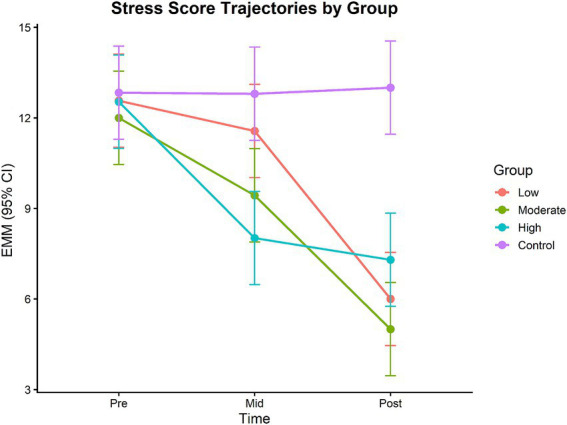
Trends in estimated marginal means of stress at different time points.

The findings for stress provided further support for this overall pattern (Figure 3). Stress was the only dimension that showed clear between-group differences as early as midtest. The high-intensity group scored significantly lower than the low-intensity and control groups, and the moderate-intensity group also scored lower than the control group. This suggests that stress may be more sensitive to moderate- and high-intensity aerobic running in the early stage of the intervention. However, by posttest, the overall pattern was again similar to that observed for depression and anxiety. All three exercise groups showed significantly lower stress scores than the control group, whereas no significant differences were found among the exercise groups. The within-group results showed a similar pattern. The moderate-intensity group improved steadily across all time intervals. The high-intensity group showed clear early improvement, but later gains were limited. The low-intensity group showed a delayed but meaningful benefit. Therefore, the stress findings also point to differences in trajectory across exercise intensities rather than endpoint superiority. Compared with depression and anxiety, direct exercise-based evidence on stress outcomes in adolescents with PIU remains limited. In this sense, the present study adds to the literature by showing how stress changed over time under different exercise intensities. It is also worth noting that Wang et al. (2026) found, in a meta-analytic structural equation model of problematic smartphone use, that physical activity may reduce problematic use mainly through lower negative emotion, especially depression and stress, and through better self-control. Although that study did not focus on adolescents with PIU, its mechanistic findings are still relevant to the present emphasis on changes in depression and stress.

The findings may reflect differences in how adolescents experienced and tolerated the training load over repeated sessions. From a dual-mode perspective, affective responses to exercise may vary across intensity levels because cognitive appraisal and interoceptive cues may be weighted differently as intensity changes (Ekkekakis et al., 2008; Ekkekakis, 2009). In this sense, the present pattern may suggest a psychobiological “sweet spot,” in which exercise is challenging enough to promote engagement and emotional improvement, but not so aversive or fatiguing that it reduces tolerability or sustained participation (Williams et al., 2008). In the current sample, moderate-intensity running may have come closer to such a balance. By contrast, the relatively higher-intensity condition appeared to produce earlier improvement but less additional gain later, whereas the low-intensity condition may have required a longer exposure period before measurable benefits emerged. In addition, the social and behavioral context of exercise may also matter. For adolescents with PIU, regular exercise may provide structure, daily routine, a sense of mastery, and opportunities for positive social involvement (Doré et al., 2020). These features may be especially relevant for adolescents who often experience difficulties in emotion regulation, self-control, and daily functioning (Liu et al., 2024). From this perspective, the observed differences across intensity levels may be related not only to training load itself, but also to how each intensity shapes perceived challenge, engagement, and the likelihood of maintaining participation over time (Williams et al., 2008). These interpretations remain tentative and should be tested directly in future studies using measures such as affective response, perceived exertion, self-efficacy, and adherence.

The present findings also have several practical implications. Most importantly, schools, families, and communities do not need to rely only on high-intensity exercise when trying to improve emotional health in adolescents with PIU. All three exercise intensities showed beneficial effects relative to the control condition. This is also consistent with the conclusion of Zhou Z. et al. (2024) that sport-based intervention is among the most promising single approaches for adolescent internet addiction. At the same time, the different trajectories across intensities suggest that exercise prescriptions can be tailored to specific intervention goals. If the main goal is to obtain faster short-term improvement, especially in anxiety or stress, high-intensity running condition may be useful when adequate supervision and safety can be ensured. If the main goal is to promote a more continuous pattern of emotional improvement over time, moderate-intensity aerobic running may represent a reasonable starting option. Low-intensity exercise should also not be overlooked, because it still showed significant benefits at posttest. It may be a more acceptable starting point for adolescents with lower fitness, lower confidence, or weaker exercise adherence. Thus, the practical value of the present findings lies not in identifying a single “best” intensity, but in showing that different intensities may be matched to different intervention priorities.

This study also has several limitations. First, the sample was drawn from a specific adolescent population, which may limit the generalizability of the findings to other age groups, cultural contexts, or clinical populations. Second, the intervention lasted 12 weeks, but no long-term follow-up was conducted. Therefore, it remains unclear whether these improvements would be maintained after the intervention ended. Third, the study relied mainly on self-report measures. Although these tools are practical and psychometrically acceptable, they may not fully capture clinical severity or subtle changes in emotional functioning. Fourth, physiological and neurobiological indicators were not included. As a result, the potential mechanisms underlying the different time patterns across exercise intensities could not be tested directly. Fifth, the study used a blank control rather than an active control group. In addition, the actual execution of exercise intensity, training adherence, and individual exercise dose were not monitored objectively. Therefore, the interpretation of intensity-specific effects should remain cautious. Sixth, although participants were assigned to different intervention conditions, individual differences in baseline fitness, motivation, sleep, family support, and adherence may also have influenced emotional outcomes. Future studies should include longer follow-up periods, multi-source or objective measures, and process-related indicators to clarify the long-term effects and underlying pathways of exercise-based emotional improvement in adolescents with PIU. Finally, the upper exercise zone in this study (60%–69% HRmax) did not correspond to conventionally defined vigorous intensity. Thus, the present findings should be interpreted as evidence from study-defined relative intensity levels in physically inactive adolescents with PIU, rather than from a truly vigorous or HIIT-type protocol. Different emotional and physiological response patterns may emerge under a higher-intensity design.

Overall, the present findings show that aerobic running can alleviate depression, anxiety, and stress in adolescents with PIU, and that different exercise intensities may lead to different trajectories of improvement. The findings do not support a simple hierarchical view that the highest intensity always produces the greatest benefit. Instead, they suggest a more differentiated pattern: high-intensity exercise may promote faster early improvement, moderate-intensity exercise may produce more stable and sustained change, and low-intensity exercise may still be meaningful despite a slower onset. This conclusion is consistent with previous evidence that exercise interventions are generally effective, and it also adds new evidence on when improvement appears and how it develops under different exercise intensities. This more differentiated interpretation has value in both theory and practice, because it suggests that exercise intensity should be judged not only by final outcomes, but also by the dynamic process through which emotional benefits unfold over time.

## 5 Conclusion

This study examined the effects of a 12-week aerobic running intervention at low, moderate, and high intensities on depression, anxiety, and stress in adolescents with PIU. All three exercise conditions produced significantly lower posttest scores than the control condition across the three emotional outcomes. However, no statistically significant posttest differences were observed among the three exercise groups. The main difference across intensities was the pattern of change over time: the relatively higher-intensity condition was associated with earlier improvement, the moderate-intensity condition with a more continuous pattern of within-group improvement, and the low-intensity condition with later-emerging benefit. Overall, the findings suggest that aerobic running is a useful intervention for adolescents with PIU and that intensity-related differences are better understood in terms of trajectory and timing rather than endpoint superiority. From a practical perspective, exercise prescriptions for this population may be tailored according to intervention goals, individual tolerance, and program context.

## Data Availability

The original contributions presented in the study are included in the article/supplementary material, further inquiries can be directed to the corresponding author.
